# Evaluation of FindMyApps: protocol for a randomized controlled trial of the effectiveness and cost-effectiveness of a tablet-based intervention to improve self-management and social participation of community-dwelling people with mild dementia, compared to usual tablet use

**DOI:** 10.1186/s12877-021-02038-8

**Published:** 2021-02-24

**Authors:** David Peter Neal, Yvonne J. F. Kerkhof, Teake P. Ettema, Majon Muller, Judith Bosmans, Evelyn Finnema, Maud Graff, Karin Dijkstra, Max L. Stek, Rose-Marie Dröes

**Affiliations:** 1 Department of Psychiatry, Amsterdam University Medical Centers, location VUmc, Amsterdam, Netherlands; 2 Saxion University of Applied Sciences, Deventer, Netherlands; 3 Department of Internal Medicine, Amsterdam University Medical Centers, Amsterdam, Netherlands; 4 Department of Health Sciences, Vrije Universiteit Amsterdam, Amsterdam, Netherlands; 5 University Medical Centre Groningen, Groningen, Netherlands; 6 Radboud University Medical Centre, Nijmegen, Netherlands; 7 Department of Old Age Psychiatry, GGZ inGeest, Amsterdam, Netherlands

**Keywords:** Dementia, Social health, Psycho-social care, Quality of life, Digital health technology

## Abstract

**Background:**

For the rising number of people living with dementia, cost-effective community-based interventions to support psychosocial care are needed. The FindMyApps intervention has been developed with and for people with dementia and their caregivers, to help them use tablets to facilitate self-management and engagement in meaningful social activities. A feasibility study and exploratory pilot trial evaluating FindMyApps have been carried out. This definitive trial further evaluates the effectiveness of the intervention and, for the first time, the cost-effectiveness.

**Methods:**

A randomized controlled non-blinded single-center two-arm superiority trial will be conducted. Community-dwelling people with Mild Cognitive Impairment (MCI), or dementia with a Mini Mental-State Examination (MMSE) of > 17 and < 26, or Global Deterioration Scale 3 or 4, with an informal caregiver and access to a wireless internet connection will be included. In total, 150 patient-caregiver dyads will be randomly allocated to receive either usual care (control arm – tablet computer; *n* = 75 dyads) or usual care and the FindMyApps intervention (experimental arm – tablet computer and FindMyApps; n = 75 dyads). The primary outcomes are: for people with dementia, self-management and social participation; for caregivers, sense of competence. In addition to a main effect analysis, a cost-effectiveness analysis will be performed. In line with MRC guidance for evaluation of complex interventions a process evaluation will also be undertaken.

**Discussion:**

Results of the trial are expected to be available in 2023 and will be submitted for publication in international peer-reviewed scientific journals, in addition to conference presentations and reporting via the EU Marie Sklodowska-Curie DISTINCT ITN network. By providing evidence for or against the effectiveness and cost-effectiveness of the FindMyApps intervention, the results of the trial will influence national implementation of FindMyApps. We hope that the results of the trial will further stimulate research and development at the intersection of technology and psycho-social care in dementia. We hope to further demonstrate that the randomized controlled trial is a valuable and feasible means of evaluating new digital technologies, to stimulate further high-quality research in this growing field.

**Trial registration number:**

Netherlands Trial Register: NL8157; registered 15th November 2019.

## Background and rationale

In the Netherlands, 280,000 people live with dementia. This number is anticipated to more than double to 620,000 in 2050 [1]. Almost three quarters live at home. Whilst many people with dementia live well and independently for many years following diagnosis, many become increasingly dependent on support from relatives as the disease progresses. Of those people living with dementia, 70% stop their daily activities due to lack of confidence, 50% avoid community interaction due to concerns about their functional limitations and 40% hardly leave their home [2, 3]. More than half of all caregivers feel heavily or very heavily burdened by caring for someone with dementia [4].

Dementia greatly impacts people’s quality of life and their social health. Social health consists of three domains: capacity to fulfill one’s potential and meet obligations; ability to manage one’s own life to engage in meaningful activities; and ability to participate in social groups [5, 6]. Health and quality of life outcomes for people with dementia depend on many factors [6–10]: personal factors such as personality, competencies and skills; disease factors, such as specific disabilities and comorbidities; social factors such as competence of caregivers, care needs or living situation; and environmental factors such as living in a non-stigmatizing, dementia-friendly community, or the availability of assistive technologies [11, 12]. Strategies to optimize the influence of these factors are almost certain to improve the social health and quality of life of people living with dementia and their caregivers, which can support people living for longer in their own home [6, 13].

eHealth interventions may be able to play a role in improving social health in dementia. The World Health Organization report, *Dementia: a public health priority*, cites examples of the use of eHealth interventions [10]. The Dutch Dementia Care Standard also specifically identifies the appropriate implementation of eHealth interventions as having the potential to support people with dementia to live in their own homes longer and in better health [14]. Millions of applications (apps) for hand-held touch screen devices (including smartphones and tablets) exist, that have been designed to help people engage in pleasurable and meaningful activities, manage everyday life and maintain their social networks. There is growing evidence that apps also have potential in supporting people with mild dementia for the same purposes [15–18]. Examples of relevant apps available in the Netherlands include Ongehinderd (which allows users to search for accessible and disability-friendly locations and activities in the Netherlands), FaceTime (which for users of Apple devices allows them to conduct video calls), and a range of brain-training apps and games [19, 20]. Such apps may have the most impact in the early stages of dementia, although with respect to computerized cognitive training, it should be noted that there is no high-quality evidence from randomized controlled trials for benefits to activities of daily living or quality of life in MCI or dementia [21, 22]. Professional care is often not offered or accepted in the early phases of dementia, yet people will already be facing significant challenges in managing daily life [23].

However, it has also been noted that there are significant challenges to the implementation of eHealth interventions in general, and tablet-based interventions specifically, for people with dementia. Many people living with dementia are unfamiliar with the use of tablets or with how to download and use tablet-based apps, and only certain apps are sufficiently user-friendly for people with dementia [24, 25]. A necessary prerequisite to realizing the potential value of usable and suitable apps would be to support people in finding apps that meet their individual needs and preferences [15].

The FindMyApps intervention was designed specifically to meet that need, in co-creation between end users (people with dementia and their caregivers), Saxion University of Applied Sciences, Eumedianet, VUmc and Radboud UMC. The development process for FindMyApps has been fully described elsewhere in the literature [26–28]. The process began with identifying the needs of those with dementia and their caregivers, following which a user-centered design process resulted in a prototype that then underwent feasibility testing and was subject to a pilot randomized controlled trial. The intervention comprises three components: training in the use of a tablet and tablet-based apps, which is based on the ‘Errorless Learning’ method, the FindMyApps app and a cloud-hosted database of user-friendly apps. The FindMyApps app functions as a personalized selection tool: people with dementia (and their caregivers) can use the app to search the database for apps for self-management and meaningful activities that fit their needs, abilities and interests. The feasibility of the FindMyApps intervention has been tested on a small scale but the effectiveness in promoting self-management and social participation, as essential components of good social health, compared to the impact of a standard tablet has not yet been investigated. Through this randomized controlled trial, we will evaluate the effectiveness of FindMyApps. In line with the Medical Research Council framework for the evaluation of complex interventions, we will also carry out a process evaluation, to understand factors that facilitate or impede the implementation of FindMyApps [29]. Finally, we will carry out a cost-effectiveness analysis and compare the cost-effectiveness of FindMyApps to published analyses of other interventions designed to improve self-management and social participation.

This publication is based on version 2.1 of the study protocol, agreed as of 14-05-2020.

## Objectives

This RCT follows feasibility and exploratory pilot studies into FindMyApps, as a next phase in the Medical Research Council’s (MRC) framework for the evaluation of complex interventions.

The objectives of this study are:

1. To evaluate the effectiveness of FindMyApps, compared to usual care (tablet use without FindMyApps), in an RCT with respect to the following outcomes:
  Persons with dementia: (primary outcomes) ability to self-manage and social participation, (secondary outcomes) experienced autonomy, neuropsychiatric symptoms and quality of life (health related);
  Caregivers: (primary outcome) sense of competence, (secondary outcomes) attitudes to dementia, experience of providing care and quality of life (health related).
2. To evaluate the cost-effectiveness of FindMyApps compared to usual care/tablet use without FindMyApps.
3. To conduct a process evaluation of the implementation of the FindMyApps intervention in the context of this study, in line with the MRC Framework for process evaluation [30], to identify factors relating to context, implementation and mechanisms of impact (usability, learnability and adoption) that may influence the outcomes of this study.

## Trial design

This is a randomized, controlled, non-blinded superiority trial with two parallel groups and two measurements (before the start of the intervention and after three months).

Alongside the effectiveness evaluation, a cost-effectiveness analysis and a process evaluation will be carried out.

The trial is registered in the Netherlands Trial Register: NL8157.

### Study setting

The FindMyApps intervention has been developed in the Netherlands with Dutch language and cultural content. This study focuses on the implementation of FindMyApps with community dwelling people in the Netherlands with MCI, confirmed at a memory clinic, or mild dementia, and their informal caregivers. Participants will be recruited nationally through a range of health and welfare organizations. The study investigators implementing the interventions and collecting data are based at Amsterdam UMC, location VUmc.

### Eligibility criteria

Study participants must provide written, informed consent before data collection for the study takes place (see Appendix 1 for sample Informed Consent Form). Participants are assumed to have capacity to consent, unless the researcher informing the participant and documenting their consent believes that there is evidence that the person is unable to understand the information provided about the study, or to retain the information for sufficient time to weigh the information and communicate their decision.

Eligibility of dyads recruited to the study is assessed primarily based on the person with dementia or MCI. However, caregivers must also be willing and able to fully participate and support the person with dementia in the study.

People with MCI or dementia must meet the following inclusion criteria at randomization: Age ≥ 18 year; either a current diagnosis of MCI (confirmed by memory clinic), or a current diagnosis of mild dementia of any etiology; community-dwelling; has access to a wireless internet connection in their home; has a caregiver (for example a spouse, child, other relative, neighbor, etc.) who also provides consent to participate in the study, does not have a diagnosis of dementia or MCI and either co-habits with the primary participant or spends at least one hour with the primary participant, on at least two separate occasions per week, every week. We recommend that the caregiver and primary participant practice together with the tablet twice a week. The caregiver must be available to support the person with dementia or MCI if they need help. That is why a caregiver must have contact with the participant at least twice per week. If the potential participant’s diagnosis is dementia (rather than MCI) then they must have either an MMSE < 26 and > 17, measured within the last three months, or a general deterioration scale (GDS) of 3 or 4, measured within the last three months. When used for diagnostic screening, an MMSE cut-off of 24 has been associated with high sensitivity and specificity for detecting dementia [31]. In this research the MMSE is not used for diagnostic screening, but to provide an up-to-date baseline with respect to cognitive function. All participants have a pre-existing diagnosis. For the purposes of this study, we have adopted a cut-off of 25 to provide additional sensitivity, at the expense of some specificity, whilst still avoiding a ceiling effect. The MMSE is widely used to assess cognitive decline but the GDS is also a reliable method to measure cognitive decline, with an internal-consistency reliability, α = 0.88 [32].

Exclusion criteria at point of screening: current diagnosis of Primary Progressive Aphasia (PPA); blindness or sufficiently severe visual impairment to make viewing content on a tablet screen impossible; insufficient competence in reading or listening modalities of the Dutch language to understand the information provided in the informed consent process; currently participating in another interventional research study or clinical trial with the potential to confound the results of both studies.

### Interventions and procedures

Eligible participant dyads will be randomized in equal proportions between a group receiving usual care and a group receiving the FindMyApps intervention.

#### FindMyApps intervention

Those participants receiving the FindMyApps intervention will receive training in the use of FindMyApps and will have access to a tablet (either an iPad running the iOS operating system or an alternative model running the Android operating system), on which the FindMyApps app is installed. The tablet may belong to the participants or be provided as part of the intervention. We outline below the FindMyApps intervention. For further details we refer readers to the published literature [26–28].

The FindMyApps app is a selection tool designed to help people with early stage dementia or MCI, if needed with support of a caregiver, find apps for self-management and meaningful activities that fit their needs, wishes and abilities [26].

The FindMyApps selection tool consists of a native app with a specially designed user interface and a cloud-hosted database of apps that have been assessed as meeting essential criteria for usability by people with dementia and MCI [28].

The user interface consists of several pages:

- a page for setting the user-profile, whereby the users are invited to answer some questions on their preferences, needs and abilities, such as letter size, and language preferences;
- a series of pages where users can search for apps for self-management and meaningful activities, within three main categories (‘In and around the house’, ‘Social Contact’ and ‘Free Time activities’), each of which is divided into a range of subcategories;
- pages containing more detailed information about individual apps and the extent to which they meet the user’s specified preferences, with links to the app store to allow the user to download the app;
- a page (MyApps) which provides an overview of apps previously viewed in the app store (iOS) or apps already downloaded by the user (Android); and
- a page on which videos providing instructions in the use of the tablet and the FindMyApps app can be viewed.

At the beginning of the intervention period, participants will receive a brief written guide to the general use of their tablet. This will be sent by email or post, dependent on the participant’s preference. The videoconferencing software Microsoft Teams will be used for an initial videocall, during which a member of the research team will expand on the content of this brief written guide with further verbal explanations and instructions. The researcher will encourage the caregiver to demonstrate functions of the tablet to the person with dementia (turning the device on and off, charging the device, changing the volume, navigating the home screen, and starting and exiting applications). During the same call, participants will be talked through the installation and initial set-up of the FindMyApps app by the researchers. Using the Microsoft Teams screen-sharing functionality, the researcher will use pre-installed system apps and the FindMyApps app on a device comparable to the model used by the participant to demonstrate how to navigate within apps and how to operate settings within apps. Participants will have the opportunity to view a short instruction film that outlines the functions of the FindMyApps app. This film was scripted by the researchers and produced by the media team at GGZ InGeest/VUmc for the FindMyApps study. Using the Microsoft Teams screen-sharing functionality, participants will have the opportunity to practice operating the tablet and the FindMyApps app, with support from the caregiver and researcher as required. The duration of this entire meeting is expected to be 45–60 min.

The caregiver of the participant dyad will receive a written guide describing the ‘Errorless learning’ method and explaining how they can use this approach to further support the person with dementia to learn to use the tablet and the FindMyApps app. The ‘Errorless Learning’ method, which has been demonstrated to be an effective method for facilitating learning of new skills by people with cognitive impairment [33]. The technique focuses on learning through the strengthening of procedural memory, by allowing a person to practice a routine or sequence of steps, with errors immediately corrected by a facilitator. This guide will be sent by post or by email, depending on the participant’s preference. The contents of the guide will be further discussed, with additional verbal explanations provided by one of the researchers, during a telephone call or videocall using the software Microsoft Teams.

During the 3 months intervention period, participating dyads are advised to use the tablet together, in 7 follow-up sessions (of recommended duration 30–45 min), during which the caregiver applies the errorless learning method to train the participant in using the tablet and the FindMyApps app. After these training sessions the participants are expected to be able to use some apps independently and use the FindMyApps app, either with some help or independently. Previous pilot studies showed that downloading new apps often still requires guidance from the caregiver, even after the training sessions [33]. Participants are free to use the tablet and associated apps as much or as little as they choose.

Participants have access to both of the previously described training films through the FindMyApps app, which they can watch at any time to review the instructions received in the first face-to-face meeting. Participants are also provided with contact details by which they can consult a helpdesk run by EUMediaNet (the technical developer of the FindMyApps app), in case of questions or problems with the tablet or apps.

#### Usual care

Those receiving usual care will have access to a tablet; either an iPad running the iOS operating system or an alternative model running the Android operating system. The tablet may belong to the participants or be provided by the researchers.

At the start of the intervention period, the participants will be provided with a brief written guide to the general use of their tablet and a list of websites from which they can learn more and find suggestions for useful and user-friendly apps. This will be sent by email or post, dependent on the participant’s preference. The videoconferencing software Microsoft Teams will be used for an initial videocall, during which a member of the research team will expand on the content of this brief written guide with further verbal explanations and instructions. The researcher will encourage the caregiver to demonstrate functions of the tablet to the person with dementia (turning the device on and off, charging the device, changing the volume, navigating the home screen, and starting and exiting applications). Using the Microsoft Teams screen-sharing functionality, the researcher will use pre-installed system apps on a device comparable to the model used by the participant to demonstrate how to navigate within apps and how to operate settings within apps. Using the Microsoft Teams screen-sharing functionality, participants will have the opportunity to practice operating the tablet, with support from the researcher as required. The duration of this entire meeting is expected to be 45–60 min.

During the 3 months intervention period, participant dyads are advised to use the tablet together, so that the person with dementia/MCI can learn with support from the caregiver. Participants are provided with a link to a video, which they can watch at any time to review the instructions received in the first face-to-face meeting. This instruction film was scripted and designed by the researchers and produced by EUMediaNet for the FindMyApps study. Participants are also provided with contact details by which they can consult the study helpdesk.

### Potential harms

As a non-clinical psychosocial intervention, and on the basis of earlier feasibility and pilot studies evaluating FindMyApps, we do not anticipate that subjects are likely to experience severe adverse events.

**Fig. 1:**
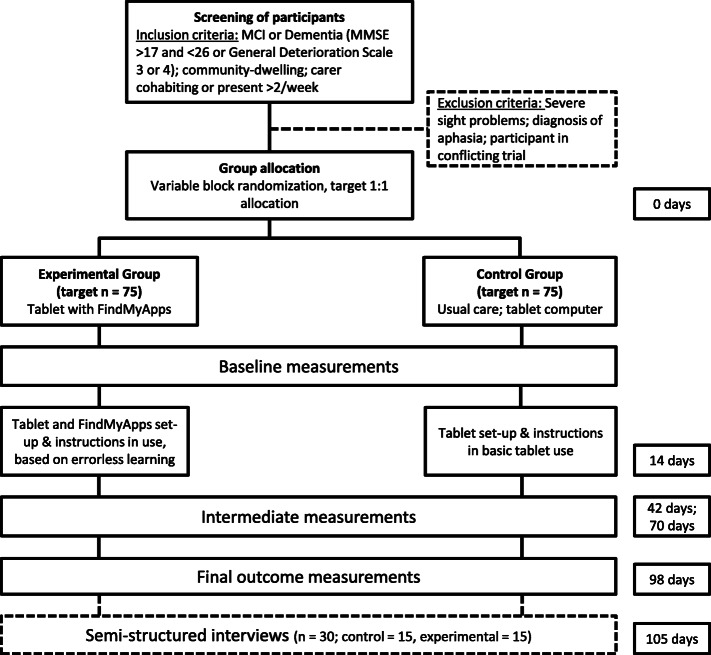
Trial flowchart

Nonetheless, study participants are advised to contact researchers at any time with any problems or adverse events, which will be logged by researchers. Researchers have contact with participants approximately every four weeks during the study (to collect intermediate app use and care use data – see Fig. 1), at which points participants are also asked about any adverse events. Logs of reported problems or adverse events will be reviewed on a regular basis and any appropriate follow-up action taken.

Special consideration will be given to avoiding and mitigating participants’ risk of exposure to the 2019-nCoV coronavirus through their participation in the study. After an initial suspension of the study, this protocol was updated on 14-05-2020, based on national guidelines to ensure the safety of participants [34]. By default, information letters and handouts will be sent to participants digitally and consent will be recorded by participants digitally, in line with guidance received from the medical ethics committee of VUmc. As such, the only remaining physical component of the intervention or method (thus representing the only increased risk to participants as a result of participation in the research) is the loaning of tablets owned by the research group to those participants who do not own a tablet.

Current advice from the World Health Organization is that the 2019-nCoV coronavirus can remain potentially infectious on surfaces for up to 72 h on plastics and stainless steel, and less than 24 h on cardboard [35]. Surface cleaning with common household products is believed to be perfectly sufficient to fully decontaminate them.

Measures will be put in place to further mitigate the risks associated with the loaning of tablets to participants. The number of members of the research team handling the tablets will be kept to a minimum. Those members of the research team handling tablets will immediately report any symptoms suggestive of COVID-19 infection and will immediately stop handling the tablets for a minimum period of two weeks.

Immediately prior to delivery of the tablet to the participant, the tablet, charging cable, box and any other physical components will be cleaned with disinfectant wipes. Tablets will be delivered to the participant’s home either by courier service or one of the research team (observing all protective public health measures), depending on what is logistically feasible. Participants will be advised not to remove the tablet from the box or handle the box for at least three days after it is delivered. If they do handle the box, when first receiving the delivery for example, they should wash their hands immediately afterwards, and before touching anything else, with soap and warm water.

At the end of the intervention period, tablets will only be collected from participants who report that they have been free of symptoms associated with COVID-19 infection for at least 14 days. Arrangements will be made for tablets to be collected either by courier service or one of the research team (observing all protective public health measures), depending on what is logistically feasible. On receipt of the tablet by the research team, the tablets and all associated components will be immediately cleaned with disinfectant wipes. No tablet will be loaned out to a new participant, within 7 days of collection from a previous participant.

### Outcomes

All primary and secondary outcomes will be assessed after three months, compared to baseline.

#### Primary outcomes

Person with dementia/MCI: Self-management; social participation; Caregiver: Sense of competence.

#### Secondary outcomes

Person with dementia/MCI: Experienced autonomy; quality of life (health related); neuropsychiatric symptoms.

Caregiver: Attitudes towards dementia; experience of providing care; quality of life (health related).

### Participant timeline

**Fig. 2:**
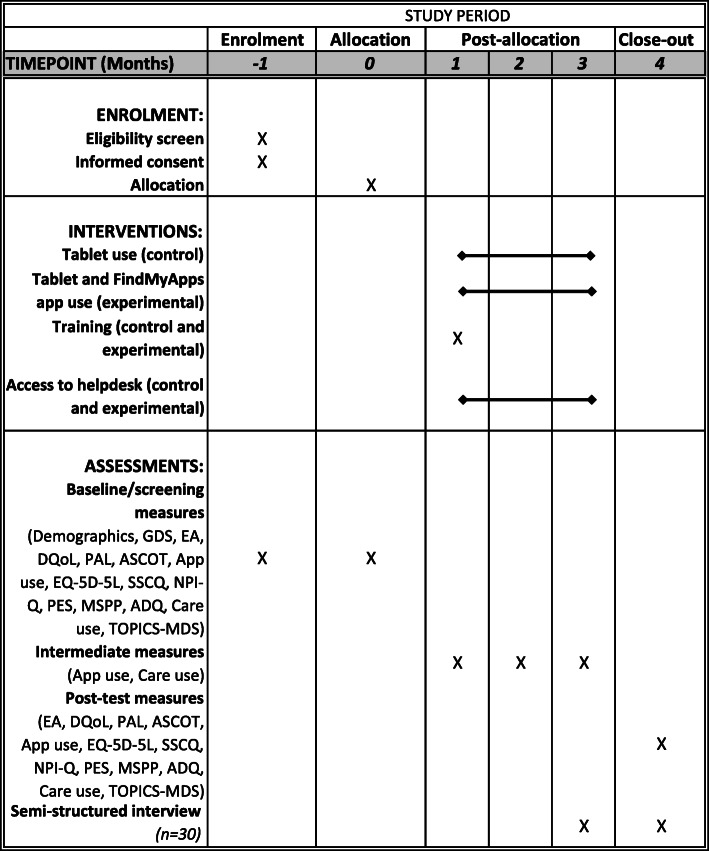
Participant timeline as per SPIRIT guideline

Figure 2 provides an overview of the time points in the study, and which data are collected and interventions delivered at each time point. Baseline measurements will be completed via online and telephone questionnaires within 7–14 days of group allocation. The first videocalls for initial set-up of the tablet and training in the use of the tablet will normally follow within 7 days of baseline measurements, and in any case will take place within 14 days of the baseline measurement.

Primary and secondary outcomes will be measured three months (84 days) after the videocall in which the tablet training is provided to the person with dementia. Semi-structured interviews (SSIs) with selected dyads will take place within 3 months (91 days) of the videocall in which the tablet training is provided to the person with dementia. Cost-effectiveness of the FindMyApps intervention will also be assessed as part of this study. Use of health and social care will be assessed on a monthly basis (after 42 and 70 days) during the three-month intervention period. Data regarding use of the tablet, which supports the process evaluation, will also be measured at intermediate points (after 42 and 70 days), as well as continuously in the experimental group by analytics software.

### Sample size

Two pilot randomized controlled trials preceded this definitive trial, both of comparable design with comparable outcome measures [36, 37]. Values of partial eta squared with respect to primary outcomes measured with instruments which will also be used in the present study, were found to range from 0.01 (MSPP) to 0.38 (PAL). These correspond to small to large effect sizes, respectively, when compared to Cohen’s suggested benchmarks [38]. Previous evaluations of interventions implemented in the Netherlands to improve social health in dementia have also found moderate to large effect sizes, with respect to similar outcome measures [39, 40]. Whilst both pilot studies also revealed small between group differences with respect to a number of outcomes, this trial will not be powered to detect small effects of the intervention. We do not believe that small effects of the intervention – even if statistically significant – would be ‘clinically’ significant. We therefore do not feel that we could justify the additional use of public funds to conduct the trial in such a way as to have adequate power to detect small effects of the intervention.

Effects of the intervention on primary outcomes for the primary participants (social participation and self-management) will be tested using MANCOVA. An a priori power calculation was conducted using the software G*Power, version 3.1, for main effects MANOVA, assuming two dependent variables, two groups, alpha = 0.05 and power = 0.8, and a moderate overall effect. The total required sample size would be *n* = 158. This is an overestimate of the required sample size to achieve a power of 0.8 because the inclusion of covariates in the MANCOVA is expected to increase the statistical power of the test compared to MANOVA (thereby reducing the required sample size for the desired power of 0.8).

In the two pilot trials evaluating the FindMyApps intervention, drop-out rates were 12 and 37% between baseline and three-month follow-up [36, 37]. Other randomized controlled trials evaluating the effectiveness of technology for social health in dementia that we are aware of, have experienced drop-out rates from baseline to three-month follow-up of around 20% [41, 42]. We are also aware of recently-conducted RCTs involving community-dwelling participants with dementia in the Netherlands, one of which reported a drop-out rate of 12% after three months and the other reported a dropout rate of 35% after six months [39, 43]. A recent review of web-based brain health interventions designed for healthy adult participants identified trials which reported drop-out after various periods of time, with rates ranging from 2 to 52% [44]. With respect to the present study, allowing for a dropout of 20% in the calculation of statistical power for a per protocol analysis, 94 participants per group would have to be randomized. However, this number may be lower in practice if the actual dropout rate is less than 20%. This is a reasonable expectation given that both the FindMyApps intervention and the trial procedure have been improved based on the results from the pilot studies. In the first instance we will therefore aim to randomize 75 participants per group. We will review this target based on the actual drop-out rate as the trial progresses.

### Recruitment

Recruitment will take place nationally throughout the Netherlands.

Participants will be recruited: via national networks of Meeting Centers, Alzheimer Cafes, memory clinics and day centers; through the Alzheimer Centre and Centre for Elderly Care Medicine of the Vrije Universiteit Medical Centre (VUmc) in Amsterdam; and through the website and social media platforms of the study, Alzheimer Nederland and other stakeholders.

The center for geriatric medicine (COGA) and Alzheimer Center of Amsterdam UMC, location VUmc, will support recruitment from their patient populations, by providing access to existing databases of eligible patients with an interest in research and by informing newly diagnosed patients who may be eligible for the study.

Interested participants will also be able to self-refer for eligibility screening via online channels, including the project website, the website of Dutch national dementia association Alzheimer Nederland and the websites of various smaller interest groups, organizations and charities.

National recruitment will continue until the required sample size has been met. It is expected that the recruitment period will extend over 23 months (100 weeks). In a previous pilot study, which was mainly conducted in two regions in the West and East of the Netherlands, 59 dyads were recruited over 12 months. Eighteen dyads were recruited in the final 12 weeks of the inclusion period for the pilot study, once relationships with referrers and public awareness were better established. The present national study can build on the network of referrers that was developed in this pilot study. Assuming at least the same average rate of inclusions for the duration of this study, and taking into account the larger recruitment area, this should allow for sufficient inclusions in the intended period.

### Allocation

Participants will be randomly assigned to either the FindMyApps (experimental) or usual care (control) arm. Randomization will be performed via Castor EDC, following a variable blocked randomization method, with a target of 1:1 allocation and randomly selected block sizes of 4 or 6. Group allocation will be stratified on the basis of diagnosis (MCI or dementia) and any prior experience with the use of a tablet (participant self-report; ‘Yes’ or ‘No’).

### Blinding

Due to the nature of the intervention, it is not possible to blind study participants, caregivers or interviewers to the allocation status of participants and outcome measures in the study are based on participant and caregiver self-report. Efforts will be made by researchers to minimize the awareness of participants in the control group of the nature of the FindMyApps intervention. In communication by researchers with study participants, the emphasis will be placed on the evaluation of the effect of the use of tablets and apps, rather than on the evaluation of FindMyApps per se. Through these measures, it is probable that participants will remain blind to the hypotheses being tested.

Data analysis will be conducted by researchers blinded to the group identities. Independent researchers with a data management role will remove or replace identifiers from the datasets prior to analysis, namely the participant codes and the labels of the group variable.

### Data collection methods

Data pertaining to outcomes reported by people with dementia will be collected from the participant by questionnaires filled in electronically by trained researchers during an interview by videocall using the software Microsoft Teams.

Data pertaining to caregiver-reported outcomes will be collected from caregivers by self-report questionnaires, sent by email with data input directly into the study database. Where this is not possible, data will be collected by the researcher by means of an interview by phone and entered by the researcher into the electronic database.

All instruments used in this study have been validated in previous psychosocial studies. The majority were used in a pilot trial evaluating the FindMyApps intervention and proved to be usable for participants.

#### Primary outcome measures

Few instruments exist, which specifically measure self-management as a component of social health and are relevant to the target group of community-dwelling people with dementia [45]. To minimize burden on participants, efficient use of fewer instruments is desirable. Self-management as a facet of good social health in dementia has been defined as the extent to which a person is able to exert control over their daily life to engage in meaningful daily activities [6]. In this definitive trial, participants’ self-management will be assessed using the Adult Social Care Outcomes Toolkit questionnaire (ASCOT). and the Pleasurable Activities List (PAL). ASCOT consists of 9 items, with each item representing one of the following domains: control over daily life, personal cleanliness and comfort, food and drink, personal safety, social participation and involvement, occupation, accommodation cleanliness and comfort and dignity [46]. Two items represent the dignity domain. Levels in each domain define the level of need: ideal state, no needs, some needs, and high-level needs. The test-retest reliability of the Dutch translation of the ASCOT is good (intra-class correlation = 0.71; 94% CI: 0.60–0.78). The short PAL used in this study consists of 19 items, each rated on a five-point rating scale ranging from 1 = “not at all” to 5 = “very frequently” with respect to how often the participant has engaged in a given activity during the preceding month. Items 1, 3 and 6 from the ASCOT questionnaire are particularly relevant to self-management, as they represent domains of control over daily life, ability to eat and drink, and general occupational function respectively. The PAL describes the frequency with which participants engage in a range of pleasurable and meaningful daily activities. Collectively the extent to which participants self-manage their daily lives is described by these instruments.

Participants’ social participation will be primarily assessed by the Maastricht Social Participation Profile (MSPP) [47] Supplementary information regarding social activity will be collected using The Older Persons and Informal Caregivers Survey Minimum DataSet (TOPICS-MDS) social activities questions [48] and the short PAL (items 4–6, 8–10, 15, 17 and 19) [28, 49]. The MSPP measures actual social participation on four indices: consumptive participation (nine items), which is characterized as benefiting from society (for example taking a course or visiting a restaurant); formal social participation (two items), which is characterized as contributing to society (participation in clubs and volunteer work); and two sub-domains (acquaintances and family) of informal social participation (nine items), which is characterized as contributing to society, receiving or both. Intraclass correlation coefficients per domain (range 0.63–0.83) are good. The TOPICS-MDS and selected items from the PAL represent a broad range of social activities, which will provide rich supplementary information to that provided by the MSPP.

Caregivers’ sense of competence will be measured with the Short Sense of Competence Scale (SSCQ), a 7-item questionnaire, with answers given on a Likert scale from 1 (strongly agree) to 5 (strongly disagree) [50]. The calculated total score (sum of all answer scores) is a reliable measure of sense of competence (Cronbach’s alpha = 0.76).

#### Secondary outcome measures

Participants’ experienced autonomy will be measured by the Experienced Autonomy questionnaire (EA) [51]. Quality of life will be assessed by the Dementia Quality of Life instrument (DQoL) [52] and the EQ-5D-5L [53, 54]. The EQ-5D-5L is not specific to dementia but is a recognized international standard in health-related quality of life assessment and will allow for comparison of the results of this cost-effectiveness analysis with other studies. The nature and severity of neuropsychiatric symptoms experienced by people with dementia will be measured by the Neuropsychiatric Inventory Questionnaire (NPI-Q) [55]. The neuropsychiatric inventory has been chosen to compliment the assessment of social participation, as behavior and mood disturbances are known to be associated with reduced social participation [56]. Moreover, previous studies have demonstrated the potential for interventions aimed at improving social health to have a positive effect on reducing neuropsychiatric symptoms [39, 57].

Caregivers’ attitudes to people with dementia will be measured by the Dutch version of the Approaches to Dementia Questionnaire (ADQ) [58]. The ADQ has been chosen on the basis of its use in stigma research to evaluate whether the pedagogical relationship between caregiver and person with dementia impacts upon the caregiver’s perception of the person receiving care. This hypothesis emerged from qualitative research undertaken as part of an earlier pilot evaluation of FindMyApps [59]. Caregivers’ experience of providing care will be measured with the Positive Experience Scale (PES) [60]. Caregivers’ quality of life will be measured with the Dutch version of the EQ-5D-5L.

Data relating to health and social care costs will be collected from participants at one, two and three months by a retrospective questionnaire adapted from the Dutch version of the RUD-Lite instrument [61]. The adaptations focus the instrument on the most important costs associated with the target group of the present study from a societal perspective. The adapted questionnaire therefore reduces the potential burden on study participants, with the expectation of improving response rates and the amount of detail given, at the expense of negligibly valuable breadth of enquiry.

#### Process evaluation

In support of the process evaluation, data regarding the nature and subjective experience of use of the tablet, the FindMyApps app and any other apps used will be collected. All participants will self-report use via a monthly questionnaire, filled in by the researchers in a telephone interview with the caregiver. Data regarding views of and interactions with the FindMyApps app by participants in the experimental group will also be recorded digitally during the intervention period by analytics software. Recorded data with respect to the FindMyApps app will include: app start events, page views, button clicks, media viewing events, and duration of sessions using FindMyApps. Due to restrictions imposed by the Android and iOS operating systems to protect privacy of tablet users, it is not feasible in this study to collect analytics data pertaining to the use of other apps on the tablet.

A purposively selected sample of 30 dyads will participate in more detailed SSIs at the conclusion of the intervention period. The SSI is based on the MRC Framework for process evaluation [30]. These interviews aim to collect information on contextual, implementation and mechanisms of impact factors that may influence intervention outcomes. Contextual factors relate to the background characteristics of participants themselves as well as their social, financial and physical environments. Implementation factors concern how the intervention is introduced and delivered to study participants by the researchers and each other. Mechanisms of impact are the participant responses to and interactions with the interventions which mediate outcomes. The interviews will be conducted by trained interviewers via videocall, using the software Microsoft Teams. A second researcher (observer) will join the videocall to register answers to the open SSI questions and any other comments of participants. Part of the interview will be administered to the person with dementia and part to the caregiver.

### Data management and analysis

Quantitative demographic data and data pertaining to participants who withdrew from the study after randomization (dropouts) will be analyzed with descriptive statistics. Differences between the trial arms will be tested with appropriate (non) parametric differences tests (Chi2, Mann-Whitney U, Student’s t-test).

For the effect evaluation, differences between the groups on the primary outcome measures will be tested by MANCOVA on post-test data, including baseline data and potential confounders as covariates in the analysis. Diagnosis (MCI or dementia) will be included as a fixed factor. We will carry out both intention-to-treat and ‘per protocol’ analyses following completion of data collection. Where we encounter missing data as a result of participants withdrawing from the study, we will use appropriate methods to impute that data and perform sensitivity analyses accordingly. Analyses will be conducted using SPSS. We will primarily report effect sizes with calculated 95% confidence intervals, however we will also report *p*-values. We will use 2-tailed p-values with an alpha of 0.05.

Both a cost-effectiveness analysis (CEA) and a cost-utility analysis (CUA) will be performed from a societal and healthcare perspective according to Dutch guidelines [62] with a time horizon of 3 months. The aim of the economic evaluation is to relate the incremental costs of FindMyApps in comparison with usual care to the incremental clinical outcomes. The following effect measures will be included in the economic evaluation: MSPP and ASCOT (person with dementia); SSCQ (caregiver); and EQ-5D-5L (person with dementia and caregiver). Missing cost and effect data will be imputed using multiple imputation according to the MICE algorithm [63]. Rubin’s rules will be used to pool the results from the different multiply imputed datasets. Bivariate regression analyses will be used to estimate cost and effect differences between FindMyApps and usual care/tablet use while adjusting for confounders if necessary. Incremental cost-effectiveness ratios (ICERs) will be calculated by dividing the difference in the mean total costs between the intervention groups by the difference in mean effect between the intervention groups. Cost-effectiveness acceptability curves will also be estimated showing the probability that FindMyApps is cost-effective in comparison with usual care/tablet use for a range of different ceiling ratios thereby showing decision uncertainty [64].

Trained researchers will provide verbal and written information to all potential participants to inform them about the nature of the study and the inherent benefits and risks. Participants will be advised to consider the information discussed before being invited to provide written, informed consent to participate. Informed consent forms will be sent to participants by post. If they wish to participate in the study, participants will be requested to return signed informed consent forms by pre-paid post to the research team. Participation in the study is conditional on both members of the dyad providing such written consent and consent will also be an ongoing process: participants will be asked on a regular basis if they are still willing to proceed with the intervention and the study [65].

All trial data will be entered electronically and stored on a secure server, based in the European Union, via Castor EDC. All data entry screens resemble the original paper versions of the corresponding forms. To help avoid error and allow checks at the time of data entry, radio buttons are employed throughout, instead of drop-down menus. Where applicable, data validation checks are applied automatically. Access to the database is password protected and the activities that each user can undertake are limited by individual access rights.

The executive researcher will be responsible for monitoring study progress, reporting on missing data and taking necessary action to follow-up on missing data or discrepancies.

With respect to the processing of personal data, a data privacy impact assessment has been carried out for this study and will be reviewed on a regular basis. Personal data will be processed in line with relevant legislation including the General Data Protection Regulation.

All data will be stored securely. Original participant files and the key to the code linking personal details and coded study data will be stored securely in a locked cupboard to which only the FindMyApps researchers have access, in a locked office, in a secure building. Original files will be stored in numerical order and maintained for a period of fifteen years after completion of the study. Any personal data stored electronically will be kept on a secure server, in a password-protected environment to which only the researchers directly involved in administering and supervising the study have access.

Data-monitoring will be undertaken by members of the project commission, with specialist input from professional statisticians as required. Given the low risk of harm posed by FindMyApps as a non-clinical intervention, and the relatively small size of the study, an independent data-monitoring committee is not necessary or valuable. There is no interim statistical analysis planned. All study investigators will have access to full data-sets.

Audits will be conducted by research and quality assurance personnel who are independent of the study, according to the established procedures of the Amsterdam Public Health Research Institute. Audits will take place once the set-up of the trial is complete and before recruitment starts (approximately month 4); at the midpoint of the study period (approximately month 18); and after recruitment is completed, during analysis of the data (approximately month 30). Any changes to the protocol that may impact upon study participants will be submitted to the medical ethics committee of VUmc and the scientific quality committee of APH for review.

### Ancillary and post-trial care

All participants, regardless of the trial arm to which they are allocated, will be offered ongoing access to the FindMyApps selection tool after the three-month study period. Study participants who have used a tablet loaned from the research organization and who wish to subsequently purchase a tablet be offered advice about the most cost-effective ways for them to obtain a tablet for themselves.

### Dissemination policy

The results of the main effect, cost-effectiveness and process analyses will be submitted as separate articles for publication in academic, peer-reviewed journals. It is anticipated that the main trial results will be submitted for publication within 6 months of completion of data collection. The results of this trial are therefore expected to be published in 2023. Study participants will be offered the opportunity to receive a summary of the main trial results. We also anticipate sharing the results with health professionals and other researchers through presentations at relevant national and international conferences. Results of the trial, including interim reports, will also be collected as part of the routine reporting of the EU Marie Sklodowska-Curie Innovative Training Network DISTINCT, through which this trial is funded. Anonymized data collected with the TOPICS-MDS instrument will be shared with the TOPICS national database, following completion of the study.

## Discussion

Two pilot RCTs helped to establish a protocol for the evaluation of the feasibility and effectiveness of the FindMyApps intervention. Although the protocol for the present study was amended in light of the 2020 coronavirus pandemic, this study goes further than any previous study of the FindMyApps intervention in four important ways.

First, by conducting the effect evaluation in a larger study (n_tot_ = 150; 75 experimental group, 75 control group), we hope to conduct this trial with a reasonable power to detect medium effect sizes (MANCOVA: η^2^ = 0.06; power 0.80, alpha = 0.05), to be able to conduct analyses from which robust conclusions about the intervention’s effectiveness for the target group of community dwelling people living with MCI/dementia and their caregiver can be drawn. A frequent criticism of eHealth or digital health interventions has been a lack of scrutiny in the context of high quality RCTs [11]. This trial will also contribute to the growing pool of RCTs in the field of eHealth and digital health, which is needed for the field to mature.

Second, by means of a process evaluation to review implementation of the FindMyApps intervention among a larger group of participants, we aim to leverage better insight into the contextual, implementation and mechanisms of impact factors that may influence implementation and adoption of FindMyApps, and the study outcomes. This will better inform further evaluation and implementation of FindMyApps compared with existing research.

Third, we aim to extend recruitment for the study from a regional to a national level, to include participants from throughout the Netherlands. With the exception of the three characteristics on which participants are stratified prior to group allocation (diagnosis of MCI or dementia; whether the participant has previously used a tablet or not; and whether or not the caregiver cohabits with the person with dementia), there is no a priori hypothesis regarding subgroups who may derive more benefit from FindMyApps. The trial is therefore designed to be as representative and ecologically valid as is feasible within the scope of a controlled trial. By recruiting people with dementia of different etiologies and with a broad demographic base, the conclusions drawn from this trial will be applicable to a broad group of people with dementia and at a national level. This is important for the planning of large-scale implementation of the FindMyApps intervention if it proves to be effective. Regardless of the main effect and cost-effectiveness analyses, subgroup analyses in the present study may suggest hypotheses relating to effectiveness or cost-effectiveness for specific target groups, which could form the basis of future studies.

Fourth, this trial incorporates for the first time a cost-effectiveness analysis. If a main effect of the FindMyApps intervention is found, we will also be able to conduct an analysis allowing a comparison of the cost-effectiveness of FindMyApps with other interventions targeting similar outcomes in similar target groups and settings. Combined with the effect analyses, this will directly inform decision-making by policy-makers, care funders, care providers and individuals with respect to the implementation of the FindMyApps intervention, and/or other interventions which target similar outcomes. As such, the present study will support the efficient use of limited resources within the care sector in the Netherlands to improve the lives of community-dwelling people with dementia and their caregivers.

Limitations of the research will also be fully discussed alongside the publication of any results. Some limitations are inherent to the design of the study. Most importantly, it is not possible to blind the participants or the researchers administering the intervention to the group assignment. Participation in the study is voluntary and it should also be anticipated that the study population may differ from the general population, with respect to their intrinsic motivation and interest in the use of a tablet. Finally, the dyadic design of the intervention means that the results of the study will not be generalizable to the implementation of the intervention with people with dementia in the absence of input from a formal or informal caregiver.

We aim to widely disseminate the results of this study, to facilitate further improvement of FindMyApps and inform the design and implementation of other tablet-based interventions for the provision of psychosocial care for people with dementia. Through widespread reporting of the results, our goal is to reduce stigma experienced by older users of technology. We expect to provide results that will support efficient and effective decision-making by policymakers, care organizations and individuals with regard to investments in technology for older users of technology, especially those with cognitive impairments. Depending on the outcomes of the effect and cost-effectiveness analyses, we will take action to improve the intervention and/or stimulate further dissemination, if appropriate.

## Data Availability

Not applicable.

## Abbreviations

ADQ: Approaches to dementia questionnaire
APH: Amsterdam Public Health Research Institute
ASCOT: Adult social care outcomes toolkit questionnaire
DQoL: Dementia quality of life instrument
EA: Experienced autonomy questionnaire
GDS: Global deterioration scale
MCI: Mild cognitive impairment
MMSE: Mini mental state examination
MRC: Medical Research Council
NPI-Q: Neuropsychiatric inventory questionnaire
PAL: Pleasurable activities list
PES: Positive experience scale
RCT: Randomized controlled trial
SSCQ: Short sense of competence scale
SSI: Semi-structured Interview
TOPICS-MDS: The Older Persons and Informal Caregivers Survey Minimum DataSet
VUmc: Vrije Universiteit Medisch Centrum
