# Correction to: Evaluation of FindMyApps: protocol for a randomized controlled trial of the effectiveness and cost-effectiveness of a tablet-based intervention to improve self-management and social participation of community-dwelling people with mild dementia, compared to usual tablet use

**DOI:** 10.1186/s12877-021-02168-z

**Published:** 2021-04-13

**Authors:** David Peter Neal, Yvonne J. F. Kerkhof, Teake P. Ettema, Majon Muller, Judith Bosmans, Evelyn Finnema, Maud Graff, Karin Dijkstra, Max L. Stek, Rose-Marie Dröes

**Affiliations:** 1 Department of Psychiatry, Amsterdam University Medical Centers, location VUmc, Amsterdam, Netherlands; 2 Saxion University of Applied Sciences, Deventer, Netherlands; 3 Department of Internal Medicine, Amsterdam University Medical Centers, Amsterdam, Netherlands; 4 Department of Health Sciences, Vrije Universiteit Amsterdam, Amsterdam, Netherlands; 5 University Medical Centre Groningen, Groningen, Netherlands; 6 Radboud University Medical Centre, Nijmegen, Netherlands; 7 Department of Old Age Psychiatry, GGZ inGeest, Amsterdam, Netherlands

**Correction to: BMC Geriatr 21, 138 (2021)**

**https://doi.org/10.1186/s12877-021-02038-8**

Following publication of the original article [1], the author notified about the error in Funding note that was made by the Publisher. Correct Funding note should be as follows:

This project is part of the Marie Skłodowska Curie Actions Innovative Training Network H2020 MSCA ITN, under grant agreement number 813196. A grant from De Bavo Stichting will meet costs associated with a helpdesk function during the trial period. Material and other costs will be met by the project partners (Amsterdam UMC, Hogeschool Saxion and Radboud UMC), with support from the Foundation for Support VCVGZ. The FindMyApps app has been developed under contract by Dutch company EUMedianet, whilst the academic partners named above remain owners of the intellectual property, as per consortium agreement. None of the funding bodies had any role in the design of the study, nor will they have any role in the collection, analysis, or interpretation of data, nor in writing this or future manuscripts.

The publisher apologizes to the authors and readers for the error and inconvenience.
